# Injury Mechanisms, Management Patterns, and Early Outcomes After Surgical Treatment of Hand Trauma: A Franco-Romanian Two-Center Cohort Study

**DOI:** 10.3390/medsci14060627

**Published:** 2026-10-01

**Authors:** Ana-Maria Ciobanu, Florence Vigouroux Guillet, Larisa-Marina Zahan, Bianca-Ana Dmour, Bogdan Puha, Ștefan-Dragoș Tîrnovanu, Bogdan-Alexandru Enache, Mihaela-Camelia Tîrnovanu, Mihai-Codrin Constantinescu, Mihaela Pertea, Bogdan Veliceasa, Luminita Labusca, Awad Dmour

**Affiliations:** 1 Department of Orthopedic, Trauma and Hand Surgery, Centre Hospitalier du Mans, 194 Avenue Rubillard, Cedex 9, 72037 Le Mans, France; anamariamateies@yahoo.com (A.-M.C.); f.vigouroux88@gmail.com (F.V.G.); larisssa_z@yahoo.com (L.-M.Z.); 2 Department of Microbiology, “Sfânta Parascheva” Clinical Hospital of Infectious Diseases, 2 Octav Botez Street, 700116 Iasi, Romania; 3 Grigore T. Popa University of Medicine and Pharmacy, 700115 Iasi, Romania; bianca-ana.dmour@umfiasi.ro (B.-A.D.); stefan-dragos.tirnovanu@umfiasi.ro (Ș.-D.T.); enache.bogdan-alexandru@d.umfiasi.ro (B.-A.E.); mihaela.tirnovanu@umfiasi.ro (M.-C.T.); mihai-codrin.constantinescu@umfiasi.ro (M.-C.C.); mihaela.pertea@umfiasi.ro (M.P.); bogdan.veliceasa@umfiasi.ro (B.V.); dmour-awad@umfiasi.ro (A.D.); 4 Department of Orthopedic and Traumatology, Sf. Spiridon Emergency County Hospital, 700111 Iasi, Romania; llabusca@phys-iasi.ro; 5 Department of Plastic Surgery and Reconstructive Microsurgery, Sf. Spiridon Emergency County Hospital, 700111 Iasi, Romania; 6 National Institute of Research and Development for Technical Physics, 700050 Iasi, Romania

**Keywords:** hand lacerations, hand trauma, postoperative antibiotics, antibiotic prophylaxis, surgical site infection, antimicrobial stewardship, wound irrigation, hand surgery

## Abstract

**Background:** Management of surgically treated hand trauma varies according to injury characteristics, referral pathways, and institutional practice, particularly regarding postoperative antibiotic therapy and wound cleansing. This study compared injury patterns, management strategies, and early postoperative outcomes between two European referral centers and evaluated reported practice through a parallel surgeon survey. **Methods:** We conducted a retrospective two-center cohort study including 360 adults undergoing surgical treatment for hand trauma between April 2024 and March 2025 in Le Mans, France (*n* = 270), and Iași, Romania (*n* = 90). Injury mechanisms, anatomical involvement, postoperative antibiotic use and duration, wound irrigation and cleansing practices, follow-up, and postoperative infection were compared. Because the cohorts differed substantially in case mix, a secondary sensitivity analysis was performed using the Romanian acute-injury subgroup (*n* = 63). A parallel anonymous survey included 70 French and 40 Romanian surgeons involved in hand trauma care. **Results:** Injury mechanisms differed between centers (*p* < 0.001), with powered-tool or machinery injuries occurring in 34.4% of Romanian and 15.6% of French patients. The Romanian cohort also had higher frequencies of tendon injury (90.0% vs. 30.7%), neurovascular involvement (57.8% vs. 14.1%), joint involvement (23.3% vs. 12.2%), and associated fracture (27.8% vs. 5.2%; all *p* ≤ 0.016). Continued postoperative antibiotics were administered to 97.8% of Romanian and 55.2% of French patients (*p* < 0.001). Among treated patients, 1- to 3-day courses predominated in Romania (85.2% vs. 16.2%; *p* < 0.001). Wound-cleansing practices also differed substantially. Among patients with documented follow-up, postoperative infection was identified in 3/90 Romanian patients (3.3%) and 3/171 French patients (1.8%; *p* = 0.418). In the Romanian acute-injury subgroup, infection occurred in 1/63 patients (1.6%). Survey responses also demonstrated variation in postoperative antibiotic duration and perceptions of existing recommendations. **Conclusions:** Substantial center-level variation was observed in case mix, postoperative antibiotic prescribing, treatment duration, and wound-cleansing practices. Few postoperative infections were documented among patients with available follow-up; however, differences in eligibility criteria, injury complexity, and follow-up completeness preclude conclusions regarding comparative treatment effectiveness. Survey findings provided complementary evidence of variability in reported practice rather than validation of the center-level clinical findings. Prospective multicenter studies using standardized injury classification and follow-up are needed to develop more consistent, risk-based approaches to antimicrobial and wound management in hand trauma.

## 1. Introduction

Hand trauma represents a frequent presentation in emergency and specialist hand surgery practice and encompasses a broad spectrum of injuries, ranging from isolated skin lacerations to complex lesions involving tendons, nerves, vessels, joints, and bone. When appropriate wound cleansing, debridement, repair, and closure are performed, postoperative infection rates are generally low, particularly in uncomplicated injuries [1,2,3,4]. However, the management of more complex injuries remains less standardized, and treatment decisions may vary substantially according to injury severity, institutional protocols, and surgeon preference.

One area of particular variability is the use of postoperative antibiotics. Current perioperative recommendations provide guidance for general surgical prophylaxis and for clearly defined high-risk situations, but they do not establish specific criteria for continued postoperative antibiotic therapy after most surgically treated hand injuries [5]. As a result, antibiotic prescribing may be influenced by the presence of deeper structural damage, contamination, fracture, patient comorbidities, local practice patterns, and perceived medicolegal concerns [6].

Most available evidence has focused on simple or clean incised hand injuries. Whittaker et al. conducted a randomized, double-blind, placebo-controlled trial including clean incised injuries involving the skin, tendons, and nerves and found no statistically significant difference in infection rates between antibiotic regimens and placebo [7]. Murphy et al. subsequently analyzed 13 studies involving 2578 patients and found no significant reduction in infection following antibiotic prophylaxis compared with placebo or no treatment (RR 0.89; 95% CI 0.65 to 1.23; *p* = 0.49) [1]. Importantly, open fractures, crush injuries, and bite wounds were excluded from that analysis, limiting the applicability of these findings to more complex forms of hand trauma.

More recently, a 2025 network meta-analysis incorporating 13 randomized trials and two non-randomized prospective studies, with a total of 3898 participants, reported a pooled surgical site infection prevalence of 3.6%. Prophylactic antibiotics did not significantly reduce infection risk compared with placebo or no antibiotic treatment. Nevertheless, the authors considered the available evidence inconclusive because of low event rates, wide confidence intervals, and moderate-to-high risk of bias in many included studies [3]. These limitations are particularly relevant when considering injuries with associated tendon, neurovascular, joint, or osseous involvement, for which evidence remains comparatively sparse.

Variation in management extends beyond antibiotic prescribing. Wound irrigation and cleansing practices also differ between surgeons and institutions, including the choice of saline, antiseptic solutions, and adjunctive wound-cleansing agents. Such differences may reflect local protocols and clinical tradition as much as comparative evidence. Because wound management and antimicrobial therapy are closely linked in the prevention of postoperative infection, evaluating these practices together may provide a more complete representation of real-world hand trauma care.

At the same time, unnecessary or prolonged antibiotic exposure must be considered within the broader context of antimicrobial stewardship. Responsible antimicrobial use aims to limit avoidable treatment while preserving clinical effectiveness and reducing the selective pressure that contributes to antimicrobial resistance [8]. Antibiotics may also expose patients to adverse drug reactions, including allergic reactions, without a clearly demonstrated benefit in lower-risk settings [9]. Accordingly, both the decision to prescribe postoperative antibiotics and the duration of treatment should be interpreted in relation to injury complexity and the wider surgical management strategy.

The present study aimed to compare injury mechanisms, anatomical injury patterns, perioperative management, postoperative antibiotic prescribing and treatment duration, wound irrigation and cleansing practices, and early postoperative outcomes among patients undergoing surgical treatment for hand trauma at two European referral centers. A parallel surgeon survey was used to further characterize variation in antibiotic prescribing and wound-management practices.

## 2. Materials and Methods

### 2.1. Study Design and Setting

We conducted a retrospective, observational, international two-center cohort study including adult patients who underwent surgical treatment for hand trauma at two European referral centers: SOS Main, Centre Hospitalier du Mans, Le Mans, France, and the Plastic and Reconstructive Surgery service of “Sf. Spiridon” County Clinical Emergency Hospital, Iași, Romania. The study period extended from April 2024 to March 2025.

Data were retrospectively extracted from emergency department records, operative reports, inpatient documentation, postoperative consultation notes, and electronic medical records.

The organization of hand trauma care differed between the two settings. The French cohort originated from a dedicated SOS Main service specializing in hand trauma. At the Romanian participating center, hand trauma is managed within the Plastic and Reconstructive Surgery service, within a healthcare setting in which both plastic and orthopedic surgeons may manage hand injuries according to local organization, referral pathways, and available expertise. These differences in service organization were considered potentially relevant to the case mix encountered at each center and were taken into account when interpreting between-center comparisons.

### 2.2. Participants and Data Collection

Patients were eligible if they were aged 18 years or older and underwent surgical treatment for a traumatic injury involving the hand. Injuries affecting the skin and soft tissues, flexor or extensor tendons, nerves, vessels, joints, or bone were included.

The study included acute lacerations, crush and avulsion injuries, open injuries associated with fractures, tendon or neurovascular lesions, joint injuries, and selected secondary procedures related to previous hand trauma.

Bite injuries were excluded because of their distinct microbiological characteristics and antimicrobial treatment indications. Patients with an established infection at the time of presentation, including cellulitis, abscess formation, purulent wound infection, or an infected neglected wound, were also excluded. Injuries involving a retained foreign body requiring specific treatment were not included. Patients undergoing surgery for non-traumatic hand conditions were excluded.

The two cohorts were not assembled using identical eligibility frameworks. The French dataset available for the present analysis consisted of 270 patients who had fulfilled the eligibility criteria of the original acute hand-laceration cohort. That cohort primarily included surgically treated acute hand injuries and used a more restrictive selection framework, including exclusion of fractures requiring osteosynthesis, heavily contaminated wounds, established infection at presentation, selected complex reconstructive injuries, and delayed surgical management.

At the Romanian center, the institutional source database contained 381 admissions during the corresponding study period. Diagnostic, procedural, and operative documentation was screened to identify adult patients undergoing surgical treatment for traumatic hand injuries, followed by patient-level review. Ninety patients were retained for the present analysis. The Romanian cohort consequently represented a broader spectrum of traumatic hand pathology, including acute injuries, more complex reconstructive injuries, associated fractures requiring fixation, and selected delayed or secondary post-traumatic procedures.

### 2.3. Data Collection and Definitions

The following variables were collected: age, sex, documented diabetes mellitus, obesity, smoking status, tendon injury, nerve injury, vascular injury, neurovascular involvement, joint involvement, associated fracture, timing of surgical treatment, postoperative antibiotic administration, duration of antibiotic treatment, wound irrigation and cleansing practices, postoperative follow-up, postoperative infection, infection-related readmission, and surgical reintervention.

Tendon injury was defined as traumatic involvement of a flexor or extensor tendon. Neurovascular involvement was defined as injury to a peripheral nerve and/or arterial structure. Joint involvement was defined as traumatic communication of the wound with an interphalangeal or metacarpophalangeal joint. Associated fracture was recorded whenever an osseous injury accompanied the hand trauma, irrespective of whether surgical fixation was required.

Injury mechanism was determined from the documented circumstances of injury and classified into four mutually exclusive categories: sharp or cutting injury, powered tool or machinery injury, crush, avulsion or entrapment injury, and other or unknown mechanisms. When more than one mechanism descriptor was present, classification was based on the primary mechanism responsible for the hand injury.

Diabetes mellitus was considered documented when identified by diagnostic coding or explicitly recorded in the medical record. In the absence of either, diabetes was classified as not documented. Smoking status and obesity were recorded from available clinical documentation. Patients with unknown or insufficiently documented status were excluded from the corresponding denominator.

Timing information was reviewed from the available clinical and operative records to identify delayed presentations and secondary post-traumatic procedures. However, exact injury-to-presentation and presentation-to-surgery intervals were not documented with sufficient completeness and consistency across both centers to permit reliable quantitative comparison. Delayed or secondary presentations were therefore classified clinically from the available documentation rather than analyzed using continuous time intervals.

### 2.4. Classification of Injury Complexity

For the principal analysis, all patients were analyzed according to the center at which treatment was performed.

To further characterize differences in injury severity, the Romanian cohort was additionally classified into three clinical groups: acute injuries, acute complex injuries, and delayed or secondary post-traumatic presentations.

Acute complex injuries included injuries requiring more extensive reconstruction, fixation of associated fractures, treatment of major soft-tissue loss, or management of severe crush or avulsion trauma. Delayed or secondary presentations included procedures undertaken for post-traumatic sequelae, secondary tendon pathology, tissue necrosis, or delayed reconstruction. The Romanian acute-injury group was also used for a secondary sensitivity analysis with the French cohort.

The complete Romanian cohort was retained in the primary analysis to characterize the full spectrum of surgically treated hand trauma and routine management encountered at the participating Romanian center during the study period. Accordingly, the primary between-center analysis was intended to describe differences in case mix and management patterns rather than to compare equivalent clinical populations or estimate treatment effects. Because the Romanian cohort included complex and delayed or secondary presentations that were not represented to the same extent in the French cohort, a secondary sensitivity analysis was performed using the Romanian acute-injury subgroup.

### 2.5. Surgical Management and Wound Irrigation

Surgical treatment followed the routine principles of traumatic hand surgery at both institutions. Management included wound exploration, excision of devitalized tissue when necessary, removal of visible contamination, irrigation, assessment of injured anatomical structures, repair or reconstruction when indicated, wound closure or soft-tissue coverage, and sterile dressing.

Normal saline was used as the principal irrigation fluid at both centers. Additional wound-cleansing solutions recorded included hydrogen peroxide, povidone-iodine, and a wound-cleansing solution containing hypochlorous acid and sodium hypochlorite (Microdacyn60® Wound Care; Sonoma Pharmaceuticals Netherlands B.V., Herten/Roermond, The Netherlands). At the Romanian center, more than one adjunctive solution could be documented for the same patient. Irrigation practices were analyzed according to the solutions documented in the medical record.

### 2.6. Postoperative Antibiotic Treatment

Perioperative antibiotic prophylaxis and postoperative antibiotic treatment were recorded separately. A single antibiotic dose administered preoperatively or intraoperatively without subsequent postoperative doses was classified as perioperative prophylaxis only. Continued postoperative antibiotic treatment was defined as systemic antibiotic administration after surgery beyond this single perioperative dose.

Among patients receiving continued postoperative antibiotics, the initial postoperative treatment duration was categorized as 1 to 3 days, 4 to 7 days, or more than 7 days.

Antibiotic selection and treatment duration were determined by the treating surgeon according to the clinical characteristics of the injury and institutional practice.

In routine clinical practice, amoxicillin/clavulanate was the principal postoperative antimicrobial agent at both centers. At the French center, clindamycin was generally used in patients with beta-lactam allergy, while pristinamycin was used by some surgeons as an alternative. At the Romanian center, amoxicillin/clavulanate was frequently combined with gentamicin, with metronidazole added in selected markedly contaminated wounds; ceftriaxone was occasionally used for perioperative prophylaxis. Specific antimicrobial agents and the individual indications for each regimen were not systematically recorded at patient level in a sufficiently comparable manner to permit quantitative between-center analysis.

When postoperative infection subsequently required therapeutic antibiotic treatment, this course was recorded separately from the initial postoperative regimen. Therapeutic extension of antibiotics for an established infection was therefore not included when determining the duration of the initial postoperative antibiotic strategy.

### 2.7. Follow-Up and Postoperative Outcomes

Postoperative follow-up and infection outcomes were assessed using outpatient postoperative consultations, subsequent hospital documentation, readmission records, and the institutional electronic medical record. Patients without a documented postoperative consultation or subsequent postoperative outcome record were classified as lost to follow-up and were not assumed to be infection-free. The principal early postoperative outcome was clinically documented postoperative infection requiring systemic antibiotic treatment, surgical reintervention, or both. Infection was considered postoperative only when it developed following the index surgical procedure and was distinct from infection present at initial presentation.

Surgical reintervention for infection and infection-related hospital readmission were analyzed separately. Procedures performed for hardware removal, tendon adhesions, secondary tendon pathology, post-traumatic sequelae, or other noninfectious indications were not classified as infection-related reinterventions.

### 2.8. Statistical Analysis

Continuous variables were summarized as mean and standard deviation (SD), while categorical variables were presented as frequencies and percentages. Available denominators were reported when data were incomplete.

Clinical characteristics and management patterns were first compared between the complete French and Romanian cohorts. Age was compared using Welch’s independent-samples *t*-test. Categorical variables were compared using two-sided Fisher’s exact test, and crude odds ratios (ORs) with 95% confidence intervals (CIs) were calculated for selected binary comparisons.

Postoperative antibiotic duration was compared between centers using the predefined categories of 1 to 3 days, 4 to 7 days, and more than 7 days. The overall distribution was assessed using Pearson’s chi-square test. A secondary comparison of short-course treatment, defined as 1 to 3 days, versus treatment of at least 4 days was performed using Fisher’s exact test.

Because postoperative follow-up was incomplete in the French cohort, infection rates were compared using only patients with documented follow-up. A secondary sensitivity analysis compared the French cohort with the 63 Romanian patients classified as acute injuries.

The Romanian cohort was also analyzed according to the three predefined clinical categories of acute, acute complex, and delayed or secondary presentations. Differences in initial antibiotic duration across these categories were evaluated using Pearson’s chi-square test.

Multivariable analysis of postoperative infection was not performed because only six postoperative infections occurred across the study population. Between-center comparisons were therefore considered unadjusted. Furthermore, because each participating country was represented by a single center, observed differences were interpreted as center-level differences rather than independent effects attributable to country.

The distribution of injury mechanisms between centers was compared using Pearson’s chi-square test.

Statistical analyses were performed using IBM SPSS Statistics for Windows, version 27.0 (IBM Corp., Armonk, NY, USA). Missing observations were not imputed. Analyses were performed using available data. All tests were two-sided, and statistical significance was defined as *p* < 0.05.

### 2.9. Ethical Approval

The study was conducted in accordance with the Declaration of Helsinki. The French component was approved by the Ethics Group of the Centre Hospitalier du Mans, France (protocol no. 2025-014, 28 May 2025). The Romanian component was approved by the Ethics Committee of “Sf. Spiridon” County Clinical Emergency Hospital, Iași, Romania (approval no. 126, 16 September 2026).

The analysis was based on retrospectively collected, de-identified clinical information. Requirements regarding individual informed consent were applied according to the approval granted by each participating institution.

### 2.10. Surgeon Survey

In parallel with the retrospective cohort analysis, anonymous questionnaires were used to assess reported clinical practices among surgeons involved in the operative management of hand trauma in France and Romania. The French questionnaire was distributed in March 2025 to hand surgeons registered with the French Society for Surgery of the Hand. The Romanian questionnaire, using the same core questions, was distributed to surgeons involved in the surgical management of hand trauma during preparation of the present multicenter study, between August and September 2026. Participation was voluntary and anonymous.

The questionnaire assessed antibiotic administration in the emergency department, perioperative antibiotic prophylaxis, postoperative antibiotic prescribing, usual treatment duration, factors influencing antibiotic use, wound irrigation and cleansing practices, perceived adequacy of existing recommendations, and support for dedicated guidance in hand trauma. Item-specific denominators were used when not all respondents answered a given question. Survey responses were summarized as frequencies and percentages and compared between countries using Pearson’s chi-square test or Fisher’s exact test, as appropriate.

## 3. Results

### 3.1. Study Cohort, Patient Characteristics, and Injury Patterns

A total of 360 patients were included, comprising 270 patients treated at the French center and 90 at the Romanian center. Mean age was 41.3 ± 17.1 years in France and 45.8 ± 17.6 years in Romania (mean difference 4.53 years, 95% CI 0.34 to 8.73; *p* = 0.034). Documented diabetes mellitus was uncommon in both cohorts, occurring in 11 of 266 French patients (4.1%) and 2 of 90 Romanian patients (2.2%; *p* = 0.529).

The distribution of injury mechanisms differed significantly between centers (χ^2^ = 21.28, df = 3; *p* < 0.001). Sharp or cutting injuries represented 154 of 270 cases (57.0%) in France and 50 of 90 (55.6%) in Romania. Powered-tool or machinery injuries were more frequent in Romania, occurring in 31 of 90 patients (34.4%) compared with 42 of 270 patients (15.6%) in France. Crush, avulsion, or entrapment injuries accounted for 29 of 270 French cases (10.7%) and 2 of 90 Romanian cases (2.2%), while other or unknown mechanisms accounted for 45 of 270 (16.7%) and 7 of 90 (7.8%), respectively.

Anatomical injury patterns also differed significantly between centers. Tendon injury was documented in 81 of 90 Romanian patients (90.0%) compared with 83 of 270 French patients (30.7%; *p* < 0.001). Neurovascular injury was present in 52 of 90 Romanian patients (57.8%) and 38 of 270 French patients (14.1%; *p* < 0.001). Joint involvement occurred in 23.3% and 12.2%, respectively (*p* = 0.016), while associated fractures were documented in 27.8% of Romanian patients compared with 5.2% of French patients (*p* < 0.001). Results are shown in Table 1.

### 3.2. Postoperative Antibiotic Use and Treatment Duration

Postoperative antibiotic prescribing differed markedly between centers. Continued systemic antibiotic therapy beyond the perioperative period was administered to 88 of 90 Romanian patients (97.8%) compared with 149 of 270 French patients (55.2%), corresponding to an unadjusted OR of 35.73 (95% CI 8.62 to 148.12; *p* < 0.001). In the Romanian cohort, one additional patient received only a single perioperative antibiotic dose and one received no postoperative antibiotic treatment.

The duration of initial postoperative therapy also differed substantially. Treatment duration was known for 148 of the 149 French patients receiving postoperative antibiotics and for all 88 Romanian patients receiving continued postoperative therapy. In France, 24 of 148 patients (16.2%) received antibiotics for 1 to 3 days, 108 (73.0%) for 4 to 7 days, and 16 (10.8%) for more than 7 days. In Romania, 75 of 88 patients (85.2%) received treatment for 1 to 3 days and 13 (14.8%) for 4 to 7 days; no patient received an initial postoperative course exceeding 7 days. The overall duration distribution differed significantly between centers (χ^2^ = 108.63, df = 2; *p* < 0.001).

When duration was dichotomized as a short course of 1 to 3 days versus treatment lasting at least 4 days, patients at the Romanian center had substantially greater odds of receiving a short course (OR 29.81, 95% CI 14.32 to 62.06; *p* < 0.001).

Agent-level antimicrobial use was not compared quantitatively because specific drug information was not systematically captured in a comparable patient-level format at both centers.

### 3.3. Wound Irrigation and Cleansing Practices

Wound irrigation and cleansing practices differed between centers. In France, saline alone was used in 115 of 270 patients (42.6%) and saline combined with hydrogen peroxide in 143 of 270 (53.0%). Povidone-iodine was documented in 4 of 270 patients (1.5%), while Microdacyn was not used.

In Romania, saline alone was used in 10 of 90 patients (11.1%), saline with hydrogen peroxide in 30 of 90 (33.3%), Betadine-containing cleansing in 70 of 90 (77.8%), and Microdacyn in 72 of 90 (80.0%). Romanian wound-cleansing approaches were not mutually exclusive, as more than one adjunctive solution could be used in the same patient.

When each exposure was analyzed separately, saline-alone use was less frequent in Romania than in France (11.1% vs. 42.6%; OR 0.17, 95% CI 0.08 to 0.34; *p* < 0.001), as was saline with hydrogen peroxide (33.3% vs. 53.0%; OR 0.44, 95% CI 0.27 to 0.73; *p* = 0.001). Povidone-iodine/Betadine use was more frequent in Romania (77.8% vs. 1.5%; OR 232.75, 95% CI 77.06 to 702.97; *p* < 0.001). Microdacyn use also differed significantly between centers (80.0% vs. 0%; Fisher’s exact *p* < 0.001). Results are shown in Table 2.

### 3.4. Postoperative Follow-Up and Infection

Postoperative follow-up was documented in 171 of 270 French patients (63.3%) and in all 90 Romanian patients (100%; *p* < 0.001). Among the 171 French patients with documented postoperative follow-up, an exact follow-up interval was available for 155 patients. The median documented interval in these patients was 3 weeks (range, 2 to 6 weeks). Sixteen additional patients had documented postoperative assessment or outcome information without a precisely recorded follow-up interval. In Romania, 87 patients had approximately 2 months of documented postoperative follow-up, and 3 had approximately 6 weeks.

Among patients with documented follow-up, postoperative infection was identified in 3 of 171 French patients (1.8%) and 3 of 90 Romanian patients (3.3%). The difference was not statistically significant (OR 1.93, 95% CI 0.38 to 9.77; Fisher’s exact *p* = 0.418).

All three postoperative infections in the Romanian cohort were treated with a 14-day therapeutic antibiotic course, and none required surgical reintervention or infection-related readmission. Microbiological cultures were positive in all three cases, identifying methicillin-sensitive *Staphylococcus aureus* in two patients and *Klebsiella pneumoniae* in one. In France, all three documented postoperative infections required surgical reintervention. Microbiological confirmation was available in one case and identified methicillin-sensitive *Staphylococcus aureus*. Postoperative follow-up and infectious outcomes are summarized in Table 3.

### 3.5. Sensitivity Analysis and Romanian Clinical Categories

The Romanian cohort included 63 patients classified as acute injuries, 16 as acute complex injuries, and 11 as delayed or secondary post-traumatic presentations.

In the acute-injury sensitivity analysis, mean age was similar between the Romanian subgroup and the French cohort (43.4 ± 18.1 vs. 41.3 ± 17.1 years; *p* = 0.412). Documented diabetes mellitus was present in 1.6% and 4.1%, respectively (*p* = 0.474), while joint involvement occurred in 19.0% and 12.2% (*p* = 0.156).

Tendon injury remained more frequent in the Romanian acute-injury subgroup than in France (92.1% vs. 30.7%; *p* < 0.001), as did neurovascular injury (60.3% vs. 14.1%; *p* < 0.001) and associated fracture (17.5% vs. 5.2%; *p* = 0.002). Continued postoperative antibiotics were administered to 62 of 63 Romanian acute-injury patients (98.4%) compared with 149 of 270 French patients (55.2%; *p* < 0.001).

Postoperative infection occurred in 1 of 63 Romanian acute-injury patients (1.6%) compared with 3 of 171 French patients with documented follow-up (1.8%; *p* = 1.000).

Within the Romanian cohort, postoperative infection occurred in 1 of 63 acute injuries (1.6%), 1 of 16 acute complex injuries (6.2%), and 1 of 11 delayed or secondary presentations (9.1%). Continued postoperative antibiotics were used in 62 of 63 acute cases (98.4%), all 16 acute complex cases (100%), and 10 of 11 delayed or secondary cases (90.9%). The distribution of 1 to 3 days versus 4 to 7 days initial postoperative antibiotic courses did not differ significantly among the three Romanian clinical categories (χ^2^ = 2.08, df = 2; *p* = 0.354). Results are shown in Table 4.

### 3.6. Surgeon-Reported Practices

A total of 110 surgeons completed the questionnaire, including 70 respondents from France and 40 from Romania. The proportion of respondents reporting 15 to 20 years of surgical experience was similar between the surveyed groups, comprising 31 of 70 French respondents (44.3%) and 18 of 40 Romanian respondents (45.0%; *p* = 1.000). In France, 42 of 70 respondents (60.0%) practiced in a dedicated SOS Main center. In Romania, hand trauma is generally managed within broader plastic surgery or orthopedic services rather than through a dedicated SOS Main structure.

Patterns of antibiotic administration in the emergency department were similar between countries. In France, 11 of 70 respondents (15.7%) reported systematic administration, 48 (68.6%) selective administration, and 11 (15.7%) no routine antibiotic administration in the emergency department. Among Romanian respondents, the corresponding values were 7 of 40 (17.5%), 29 (72.5%), and 4 (10.0%), respectively. The overall distribution did not differ significantly between countries (*p* = 0.699).

Postoperative antibiotic duration differed significantly. Among French respondents who provided a treatment duration, 17 of 59 (28.8%) reported prescribing antibiotics for 1 to 3 days, 39 (66.1%) for 4 to 7 days, and 3 (5.1%) for more than 7 days. In Romania, 20 of 40 respondents (50.0%) reported treatment for 1 to 3 days, 15 (37.5%) for 4 to 7 days, and 5 (12.5%) for more than 7 days. The overall distribution differed significantly between countries (*p* = 0.018).

Perceptions regarding existing recommendations also differed significantly. In France, 25 of 70 surgeons (35.7%) considered current recommendations sufficient, 18 (25.7%) considered them insufficient, and 27 (38.6%) were unsure. Among Romanian respondents, 10 of 40 (25.0%) considered current recommendations sufficient, 25 (62.5%) insufficient, and 5 (12.5%) were unsure. The overall distribution differed significantly between countries (*p* < 0.001).

A need for more specific national guidance was reported by 48 of 70 French respondents (68.6%) and 37 of 40 Romanian respondents (92.5%; *p* = 0.004). Support for dedicated recommendations for hand trauma was high in both countries, reported by 64 of 70 French surgeons (91.4%) and 32 of 40 Romanian surgeons (80.0%), without a statistically significant difference (*p* = 0.134).

Irrigation practices showed greater similarity. Povidone-iodine use was reported by 36 of 70 French surgeons (51.4%) and 28 of 40 Romanian surgeons (70.0%; *p* = 0.072). Hydrogen peroxide use was reported by 25 of 68 French respondents (36.8%) and 16 of 40 Romanian respondents (40.0%; *p* = 0.838). Microdacyn use was reported by 20 of 40 Romanian surgeons (50.0%); the corresponding French survey item was not available for direct comparison. Results are shown in Table 5.

## 4. Discussion

The present study identified substantial variation in the clinical characteristics and perioperative management of surgically treated hand trauma between two European centers. The Romanian cohort included a considerably greater proportion of tendon, neurovascular, joint, and associated osseous injuries, while continued postoperative antibiotics were administered to nearly all patients. In contrast, postoperative antibiotics were prescribed more selectively in France. Treatment duration also differed, with short courses of 1 to 3 days predominating in Romania and courses of 4 to 7 days being substantially more common in France.

Few postoperative infections were documented among patients with available follow-up in either cohort. However, these findings should not be interpreted as demonstrating equivalence between centers or comparative effectiveness of either antibiotic strategy. The cohorts differed substantially in eligibility criteria and injury complexity, only six postoperative infections occurred, and follow-up was considerably less complete in the French cohort. Infections managed outside the participating French center or occurring among patients lost to follow-up may therefore have been missed.

The proportions of documented postoperative infections among patients with available follow-up were within the range reported in the broader hand trauma literature. Baldwin et al. reported a surgical site infection rate of 3.6%, while a large systematic review estimated an overall infection risk of approximately 5% following surgery for hand trauma [10,11]. Differences between published estimates are likely influenced by injury severity, contamination, fracture involvement, surgical complexity, definitions of infection, and duration and completeness of postoperative surveillance.

Previous studies have questioned the value of routine antibiotic administration in selected clean or uncomplicated hand injuries. Whittaker et al. found no statistically significant difference in infection between antibiotic and placebo groups in clean incised injuries involving skin, tendons, and nerves [7]. Similarly, the meta-analysis by Murphy et al., including 2578 patients, found no significant reduction in infection with antibiotic prophylaxis compared with placebo or no treatment [1]. Importantly, open fractures, crush injuries, and bite wounds were excluded from that analysis, limiting extrapolation to more severe forms of hand trauma.

The more recent network meta-analysis by Yusuf et al. included 13 randomized trials and two prospective non-randomized studies comprising 3898 patients and similarly found no significant reduction in surgical site infection with prophylactic antibiotics [3]. However, infection events were uncommon, and the available evidence was considered inconclusive because of wide confidence intervals and moderate or high risk of bias in many included studies. The present findings therefore add evidence regarding real-world practice variation rather than establishing the effectiveness or ineffectiveness of postoperative antibiotics.

An important observation in the current study is that antibiotic use differed not only in frequency but also in duration. Almost all Romanian patients received continued postoperative treatment, but most courses lasted only 1 to 3 days. In France, postoperative antibiotics were administered to a smaller proportion of patients, but treatment most commonly continued for 4 to 7 days. This suggests that antimicrobial stewardship in hand trauma may involve two distinct clinical decisions: whether postoperative antibiotics should be continued at all and, when they are prescribed, how long treatment should be maintained [6]. However, the present analysis captures only selected dimensions of antimicrobial stewardship. Antibiotic selection, combination therapy, antimicrobial spectrum, and patient-level indications were not recorded in a sufficiently comparable manner between centers. Accordingly, the observed differences in prescribing frequency and treatment duration should not be interpreted as a comprehensive assessment of the appropriateness or quality of antimicrobial stewardship at either institution.

The marked difference in injury profile is central to interpretation of the observed prescribing patterns. Romanian patients had substantially greater frequencies of tendon injury, neurovascular involvement, joint involvement, and associated fracture. These differences persisted, although to varying degrees, in the acute-injury sensitivity analysis. Consequently, the greater frequency of postoperative antibiotic administration in Romania cannot be attributed to institutional prescribing preference alone and may partly reflect differences in injury severity, reconstructive requirements, and perceived infectious risk.

Differences in the organization of hand trauma care may also contribute to the observed case mix. The French cohort originated from a dedicated SOS Main service, whereas hand injuries in the Romanian healthcare setting may be treated by plastic or orthopedic surgeons depending on local referral pathways and available expertise. At the participating Romanian referral center, complex hand trauma is predominantly managed within the Plastic and Reconstructive Surgery service. This difference in referral and specialty organization may partly explain the broader spectrum and greater complexity of injuries represented in the Romanian cohort.

The sensitivity analysis restricted to the Romanian acute-injury subgroup reduced some differences between the populations but did not eliminate them. Tendon and neurovascular injuries remained substantially more frequent in Romania, and continued postoperative antibiotic treatment remained nearly universal. Importantly, postoperative infection occurred in 1.6% of this Romanian subgroup and 1.8% of French patients with documented follow-up. Given the very small number of events and persistent differences in injury profile, these findings should be interpreted descriptively and cannot establish comparative effectiveness. The persistence of differences in postoperative antibiotic use within this more clinically restricted analysis indicates that management patterns remained different between centers; however, residual differences in injury complexity preclude attributing these differences to institutional practice or treatment strategy alone.

Adequate surgical treatment remains central to infection prevention in traumatic hand wounds. Careful exploration, removal of devitalized tissue and foreign material, irrigation, repair of injured structures, and appropriate wound closure all contribute to reducing bacterial burden. Pomares et al. demonstrated that surgical debridement and irrigation converted 87% of initially positive bacterial samples to negative cultures in a prospective series of contaminated hand wounds [12]. Although that population differed from the present cohort, the findings reinforce the importance of meticulous surgical management independently of antibiotic strategy.

The need for more specific guidance should not be interpreted as indicating an absence of perioperative antibiotic recommendations. Current SFAR and SPILF recommendations provide a general framework for surgical antibiotic prophylaxis, but they do not fully resolve questions regarding continued postoperative therapy after heterogeneous traumatic hand injuries [13]. In particular, evidence supporting a single perioperative prophylactic dose cannot automatically be extrapolated to justify several days of postoperative treatment. The large variation observed in both the clinical cohorts and surgeon survey therefore identifies postoperative duration as a particularly relevant target for future antimicrobial stewardship initiatives.

Wound irrigation and cleansing practices also differed substantially between centers. Saline alone and saline combined with hydrogen peroxide were more commonly documented in France, whereas Betadine-containing cleansing and Microdacyn were frequently used in Romania. Romanian irrigation practices were not mutually exclusive, and several adjunctive solutions could be used in the same patient. These findings therefore describe differences in clinical practice but do not establish the superiority of any irrigation strategy.

Hydrogen peroxide continues to be used because of its antimicrobial and mechanical cleansing properties, although its role in acute traumatic hand wounds remains uncertain. Experimental studies have demonstrated concentration-dependent toxicity to fibroblasts and impairment of cellular migration and proliferation [14,15]. Reviews of its use in orthopedic surgery have also raised concerns regarding cytotoxicity, impaired wound healing, and the potential for oxygen embolism when used in confined anatomical spaces [16,17]. These data do not establish clinical harm in the setting studied here, but they underline the need for a more clearly defined indication for its routine use.

The evidence regarding povidone-iodine is more heterogeneous. It has broad antimicrobial activity and remains widely used for skin preparation and selected wound-irrigation applications [18,19,20]. Some clinical reviews have not demonstrated an important adverse effect on wound healing, whereas experimental evidence indicates concentration-dependent cytotoxicity. Much of the available clinical literature originates from chronic wounds, arthroplasty, spinal surgery, and other settings that differ considerably from acute hand trauma. Therefore, the marked difference in povidone-iodine use observed between the participating centers should be interpreted as practice variation rather than evidence supporting one approach over another.

Normal saline remains a reasonable reference irrigation solution because it is isotonic, readily available, inexpensive, and does not expose viable tissues to an additional antiseptic agent. Nevertheless, the ideal solution, concentration, volume, pressure, and duration of irrigation remain incompletely defined. A systematic review of wound irrigation identified considerable variability across studies and emphasized that recommendations often depend on wound type and clinical context [21]. Standardized prospective studies focusing specifically on hand trauma are therefore needed before one irrigation strategy can be recommended over another.

The microbiological findings also warrant consideration. Methicillin-sensitive *Staphylococcus aureus* was identified in two of the three Romanian infections and in the single microbiologically documented French infection, while *Klebsiella pneumoniae* was isolated in the remaining Romanian case. Although the number of infections is too small for meaningful microbiological comparison, the predominance of *S. aureus* is consistent with the importance of skin flora in postoperative hand infections. The single Gram-negative isolate illustrates the heterogeneity that may occur in traumatic wounds and further supports a risk-based rather than uniform approach to antimicrobial management.

The parallel surgeon survey provided complementary context for the center-level clinical findings. Reported preferences regarding postoperative antibiotic duration showed broadly similar directional patterns to those observed in the participating centers. However, the clinical data were derived from only one center in each country, whereas survey respondents represented a broader range of institutions and clinical settings. The two data sources should therefore not be interpreted as demonstrating concordance between national practice and the participating centers, but rather as independently illustrating variability in antibiotic prescribing and perceptions of existing guidance. Opinions concerning current recommendations also differed. Romanian respondents were more likely to consider existing recommendations insufficient and more frequently reported a need for additional national guidance. Nevertheless, support for dedicated recommendations addressing hand trauma was high among surgeons in both countries. These findings are consistent with the variability previously described by Dunn et al. and suggest that antibiotic decisions continue to depend substantially on clinical judgment, institutional practice, and interpretation of limited evidence [6].

The findings have broader implications for antimicrobial stewardship. Routine postoperative treatment exposes patients to antibiotics when the absolute risk of infection may be relatively low, and the magnitude of benefit remains uncertain. Potential consequences include allergic reactions, gastrointestinal adverse effects, *Clostridioides difficile* infection, selection of resistant organisms, and unnecessary healthcare expenditure [6,8,9,22]. Antimicrobial stewardship in hand trauma should not imply withholding treatment from patients with clear indications. Rather, it should aim to identify those indications more precisely, select appropriate agents, and limit therapy to the shortest effective duration.

### Limitations

This study has several limitations. First, its retrospective design introduces the possibility of incomplete documentation, selection bias, and residual confounding. Each country was represented by a single clinical center, and center and country are therefore inseparable. The observed clinical differences should consequently be interpreted as differences between the participating centers rather than as definitive national practice patterns.

Second, the cohorts were assembled using different eligibility frameworks and differed substantially in injury profile. The French cohort represented a more selected population of surgically managed acute hand injuries, whereas the Romanian cohort included a broader spectrum of acute, complex, and delayed or secondary post-traumatic presentations. The primary comparison was therefore intended to characterize case mix and management patterns rather than compare equivalent patient populations. Although the acute-injury sensitivity analysis reduced some differences, important differences in anatomical injury complexity remained.

Third, postoperative follow-up was incomplete in the French cohort, with documented follow-up available for 171 of 270 patients, compared with all 90 Romanian patients. Infections occurring among French patients lost to follow-up or managed outside the participating center may therefore have been missed, creating potential differential outcome ascertainment.

Fourth, only six postoperative infections were documented. This limited statistical precision, produced wide confidence intervals, and prevented reliable multivariable modeling of infection risk. The absence of a statistically significant between-center difference should not be interpreted as evidence of equivalence.

Fifth, antibiotic exposure was not standardized. Specific antimicrobial agents and the clinical indications for individual regimens were not systematically recorded in a comparable patient-level format at both centers. Although routine prescribing practices could be described, quantitative comparison of individual antimicrobial regimens was therefore not possible.

Sixth, exact injury-to-presentation and presentation-to-surgery intervals could not be compared because timing information was documented differently and incompletely across the two retrospective datasets. Delayed and secondary presentations were therefore identified clinically rather than analyzed using standardized continuous time intervals.

Seventh, wound irrigation and cleansing data were not recorded using mutually exclusive categories at both centers. In Romania, individual patients could receive saline together with hydrogen peroxide, povidone-iodine, Microdacyn, or more than one adjunct. Comparisons of individual irrigation exposures therefore describe practice variation rather than comparative effectiveness.

Finally, the surgeon survey was voluntary and may be affected by selection, recall, and response bias. Respondents represented a broader range of clinical settings than the two centers included in the patient-level analysis, and reported practices may not directly reflect behavior within either participating institution. Survey findings should therefore be interpreted as complementary evidence of variability in reported practice rather than as nationally representative estimates.

## 5. Conclusions

This two-center retrospective study demonstrated substantial variation in the case mix and management of surgically treated hand trauma. Postoperative antibiotics were administered considerably more frequently at the Romanian center, where treatment was predominantly limited to 1 to 3 days, whereas French prescribing was more selective and most commonly continued for 4 to 7 days. Wound irrigation and cleansing practices also differed markedly.

Few postoperative infections were documented among patients with available follow-up. However, differences in eligibility criteria, injury complexity, service organization, follow-up completeness, and the small number of infectious events preclude conclusions regarding the comparative effectiveness of antibiotic or irrigation strategies.

The parallel surgeon survey independently demonstrated substantial variability in reported postoperative antibiotic practice and perceptions of existing guidance. Prospective multicenter studies using standardized eligibility criteria, injury classification, antimicrobial definitions, and complete follow-up are needed to identify which patients benefit from postoperative antimicrobial treatment and to define the shortest appropriate treatment duration.

## Figures and Tables

**Table 1:** Patient and injury characteristics according to treatment center.

Characteristic	Le Mans, France	Iași, Romania	Comparative Result
Patients, *n*	270	90	
Age, years, mean ± SD	41.3 ± 17.1	45.8 ± 17.6	Mean difference 4.53 years (95% CI 0.34 to 8.73); *p* = 0.034
Documented diabetes mellitus	11/266 (4.1%)	2/90 (2.2%)	OR 0.53 (95% CI 0.11 to 2.42); *p* = 0.529
Smoking	102/223 (45.7%)	10/90 (11.1%)	OR 0.15 (95% CI 0.07 to 0.30); *p* < 0.001
Obesity	38/269 (14.1%)	4/90 (4.4%)	OR 0.28 (95% CI 0.10 to 0.82); *p* = 0.013
Mechanism of injury			χ^2^ = 21.28, df = 3; *p* < 0.001
Sharp/cutting object	154/270 (57.0%)	50/90 (55.6%)	
Powered tools/machinery	42/270 (15.6%)	31/90 (34.4%)	
Crush/avulsion/entrapment	29/270 (10.7%)	2/90 (2.2%)	
Other/unknown	45/270 (16.7%)	7/90 (7.8%)	
Anatomical involvement			
Any tendon injury	83/270 (30.7%)	81/90 (90.0%)	OR 20.28 (95% CI 9.72 to 42.31); *p* < 0.001
Any neurovascular injury	38/270 (14.1%)	52/90 (57.8%)	OR 8.35 (95% CI 4.86 to 14.35); *p* < 0.001
Joint involvement	33/270 (12.2%)	21/90 (23.3%)	OR 2.19 (95% CI 1.19 to 4.02); *p* = 0.016
Associated fracture	14/270 (5.2%)	25/90 (27.8%)	OR 7.03 (95% CI 3.46 to 14.29); *p* < 0.001

**Table 2:** Postoperative antibiotic treatment and wound irrigation practices by center.

Management Variable	Le Mans, France	Iași, Romania	Comparative Result
Continued postoperative antibiotics	149/270 (55.2%)	88/90 (97.8%)	OR 35.73 (95% CI 8.62 to 148.12); *p* < 0.001
1 to 3 days *	24/148 (16.2%)	75/88 (85.2%)	
4 to 7 days *	108/148 (73.0%)	13/88 (14.8%)	
>7 days *	16/148 (10.8%)	0/88 (0.0%)	Global *p* < 0.001
Saline alone	115/270 (42.6%)	10/90 (11.1%)	OR 0.17 (95% CI 0.08 to 0.34); *p* < 0.001
Saline + hydrogen peroxide	143/270 (53.0%)	30/90 (33.3%)	OR 0.44 (95% CI 0.27 to 0.73); *p* = 0.001
Betadine/povidone-iodine use	4/270 (1.5%)	70/90 (77.8%)	OR 232.75 (95% CI 77.06 to 702.97); *p* < 0.001
Hypochlorous acid/sodium hypochlorite solution	0/270 (0.0%)	72/90 (80.0%)	*p* < 0.001

Antibiotic-duration percentages are calculated among patients receiving continued postoperative antibiotics with known treatment duration. * Percentages for antibiotic-duration categories were calculated among patients receiving continued postoperative antibiotics with known treatment duration (France, n = 148; Romania, n = 88).

**Table 3:** Postoperative follow-up and infectious outcomes according to treatment center.

Outcome	Le Mans, France	Iași, Romania	Comparative Result
Documented postoperative follow-up	171/270 (63.3%)	90/90 (100.0%)	*p* < 0.001
Postoperative infection among patients with follow-up	3/171 (1.8%)	3/90 (3.3%)	OR 1.93 (95% CI 0.38 to 9.77); *p* = 0.418
Surgical reintervention for infection	3/3 (100%)	0/3 (0%)	-

**Table 4:** Sensitivity analysis: French cohort versus Romanian acute-injury subgroup.

Characteristic	Le Mans, France	Romanian Acute-Injury Subgroup, *n* = 63	*p* Value
Age, years, mean ± SD	41.3 ± 17.1	43.4 ± 18.1	0.412
Documented diabetes mellitus	11/266 (4.1%)	1/63 (1.6%)	0.474
Any tendon injury	83/270 (30.7%)	58/63 (92.1%)	<0.001
Any neurovascular injury	38/270 (14.1%)	38/63 (60.3%)	<0.001
Joint involvement	33/270 (12.2%)	12/63 (19.0%)	0.156
Associated fracture	14/270 (5.2%)	11/63 (17.5%)	0.002
Continued postoperative antibiotics	149/270 (55.2%)	62/63 (98.4%)	<0.001
Postoperative infection among patients with follow-up	3/171 (1.8%)	1/63 (1.6%)	1.000

**Table 5:** Antibiotic prescribing practices, irrigation practices, and opinions regarding guidance among surveyed surgeons.

Survey Domain	Response	France	Romania	*p* Value
Antibiotic administration in the emergency department	Systematic	11/70 (15.7%)	7/40 (17.5%)	
	Selective	48/70 (68.6%)	29/40 (72.5%)	
	Never	11/70 (15.7%)	4/40 (10.0%)	0.699 *
Postoperative antibiotic duration	1 to 3 days	17/59 (28.8%)	20/40 (50.0%)	
	4 to 7 days	39/59 (66.1%)	15/40 (37.5%)	
	>7 days	3/59 (5.1%)	5/40 (12.5%)	0.018 *
Adequacy of current recommendations	Sufficient	25/70 (35.7%)	10/40 (25.0%)	
	Insufficient	18/70 (25.7%)	25/40 (62.5%)	
	Unsure	27/70 (38.6%)	5/40 (12.5%)	<0.001 *
Need for more specific national guidance	Yes	48/70 (68.6%)	37/40 (92.5%)	0.004
Support for dedicated hand trauma recommendations	Yes	64/70 (91.4%)	32/40 (80.0%)	0.134
Surgical experience	15 to 20 years	31/70 (44.3%)	18/40 (45.0%)	1.000
Povidone-iodine use	Yes	36/70 (51.4%)	28/40 (70.0%)	0.072
Hydrogen peroxide use	Yes	25/68 (36.8%)	16/40 (40.0%)	0.838
Microdacyn use	Yes	Not available	20/40 (50.0%)	Not tested

* Overall *p* value from Pearson’s chi-square test for the comparison of response distributions across categories. Binary comparisons were assessed using Fisher’s exact test, as appropriate.

## Data Availability

The de-identified data supporting the findings of this study are available from the corresponding author upon reasonable request and subject to institutional approval. The data are not publicly available because of privacy and ethical restrictions related to human participant information.

## Abbreviations

The following abbreviations are used in this manuscript:

CI	Confidence interval
IQR	Interquartile range
OR	Odds ratio
SD	Standard deviation
SFAR	Société Française d’Anesthésie et de Réanimation
SFCM	Société Française de Chirurgie de la Main
SPILF	Société de Pathologie Infectieuse de Langue Française
SPSS	Statistical Package for the Social Sciences
